# Impact of device resistances in the performance of graphene-based terahertz photodetectors

**DOI:** 10.1007/s12200-024-00122-6

**Published:** 2024-06-12

**Authors:** O. Castelló, Sofía M. López Baptista, K. Watanabe, T. Taniguchi, E. Diez, J. E. Velázquez-Pérez, Y. M. Meziani, J. M. Caridad, J. A. Delgado-Notario

**Affiliations:** 1 Department of Applied Physics, University of Salamanca, 37008 Salamanca, Spain; 2 Unidad de Excelencia en Luz y Materia Estructurada (LUMES), University of Salamanca, 37008 Salamanca, Spain; 3 Research Center for Electronic and Optical Materials, National Institute for Materials Science, 1-1 Namiki, Tsukuba, 305-0044 Japan; 4 Research Center for Materials Nanoarchitectonics, National Institute for Materials Science, 1-1 Namiki, Tsukuba, 305-0044 Japan; 5 Nanotechnology Group, USAL–Nanolab, University of Salamanca, 37008 Salamanca, Spain

**Keywords:** Graphene, THz, Photodetector, Field-effect transistor, Plasmonic

## Abstract

In recent years, graphene field-effect-transistors (GFETs) have demonstrated an outstanding potential for terahertz (THz) photodetection due to their fast response and high-sensitivity. Such features are essential to enable emerging THz applications, including 6G wireless communications, quantum information, bioimaging and security. However, the overall performance of these photodetectors may be utterly compromised by the impact of internal resistances presented in the device, so-called access or parasitic resistances. In this work, we provide a detailed study of the influence of internal device resistances in the photoresponse of high-mobility dual-gate GFET detectors. Such dual-gate architectures allow us to fine tune (decrease) the internal resistance of the device by an order of magnitude and consequently demonstrate an improved responsivity and noise-equivalent-power values of the photodetector, respectively. Our results can be well understood by a series resistance model, as shown by the excellent agreement found between the experimental data and theoretical calculations. These findings are therefore relevant to understand and improve the overall performance of existing high-mobility graphene photodetectors.

**Graphical Abstract:**

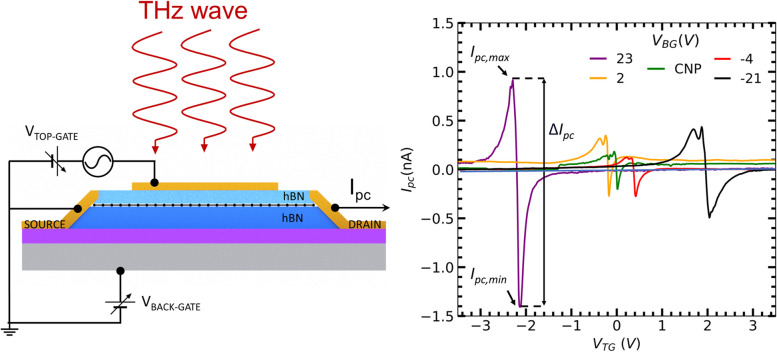

**Supplementary Information:**

The online version contains supplementary material available at 10.1007/s12200-024-00122-6.

## Introduction

Electromagnetic radiation at terahertz (THz) frequencies (0.1–10 THz) exhibit key advantageous features including a noninvasive nature (with energies of the order of few meVs) and penetration capability through opaque objects to the visible light. Such characteristics makes THz waves an amazing tool that can be used effectively in a wide variety of applications, e.g., upcoming high-speed wireless communication, medical bioimaging, information technologies, security, spectroscopy or even spintronics [1–5]. A general requirement for all of these technologies is the development of efficient photodetectors with a high sensitivity, low signal-to-noise ratio, and a fast response in the THz spectral range [6]. In recent years, the emergence of two-dimensional (2D) materials has boosted the development of novel prototype detectors and sensors operating at THz wavelengths due to their ability to exhibit striking, tunable optoelectronic properties. To date, a wide variety of 2D systems have been studied and exploited at THz wavelengths, including graphene [7, 8], transition metal dichalcogenides (TMDs) [9], black phosphorus (BP) [10], topological semimetals [11] or even MXene [12]. The capacity of these photodetectors to convert incident THz photons into electrical signals was based on different physical mechanisms, including photo-thermoelectric, bolometric, or even photogalvanic effects [7, 8, 10]. However, plasma-wave assisted mechanisms in graphene field-effect transistors (FETs) detectors (also known as plasmonic graphene FETs or plasmonic graphene photodetectors) represent the most promising route to fulfill all the required criteria to be efficiently used in real applications [13].

Up to now, it has been extensively reported how the sensitivity of plasmonic THz detectors depends strongly on several factors including the integration of external components such as i) efficient plasmonic antennas to produce optical-field enhancement [14], ii) hyper-hemispherical lenses [15] and mesoscale particles [16] to improve the coupling of the THz beam and even focusing at subwavelength frequencies, iii) the presence of asymmetry in the device structure [17] and even iv) impedances presented in read-out circuits [18–20]. Similarly, the internal material’s properties such as the mobility of free carriers in the channel [21] play a big role in the sensitivity of the device (reason why several FETs made from different materials have been studied so far [13, 22–25]). Nonetheless, the detail impact of additional (and relevant) intrinsic device parameters on the photogenerated signal at THz frequencies is less well-known in these systems.

In general, coupling of THz waves into plasmonic photodetectors based on FET architectures is effectively realized between a local (i.e., not covering the whole channel) top-gate and the source electrodes. The rectified DC current (or voltage) arising between drain and source electrodes when shining THz radiation to plasmonic photodetectors may be also heavily affected by parasitic regions in the channel (i.e., those regions in the channel not controlled by this local top-gate and not contributing in coupling the incoming THz radiation), and their impact on the overall photodetection performance of the system is not fully revealed yet. Such knowledge is important to completely understand and optimize the performance of FET devices at THz frequencies and this is the principal goal of the present study.

To do so, we have fabricated and analyzed the THz photoresponse of FET devices made from high-quality monolayer graphene, a very well-known semimetallic two-dimensional material with high-carrier mobility (> 50,000 cm^2^/(V⋅s)) even when operating at room temperature. In practice, such high-quality samples are achieved by encapsulating the monolayer between hexagonal boron nitride (hBN) [26, 27]. The device FET architecture is similar to the one used in several studies in literature reporting plasmonic photodetection [25, 28–32], having a local top-gate (TG) electrode (which does not cover the entire device channel, see experimental details below). Moreover, the presence of a global back-gate (BG) electrode in the system allows us to independently dope channel regions not affected by the top-gate, tune the access resistance of these areas and therefore study their impact on the measured THz photoresponse.

In particular, by measuring the photocurrent collected in the device at different top- and back-gate potentials, we observe regimes with a notably enhanced photoresponse (more than one order of magnitude) when the areas of the channel not affected by the top-gate are highly doped. We further demonstrate how this behavior can be fully understood by taking into account the regions with different doping levels existing in the photodetector in a series resistance model, showing an excellent agreement between this model and the experimental data.

## Results and discussion

**Fig. 1:**
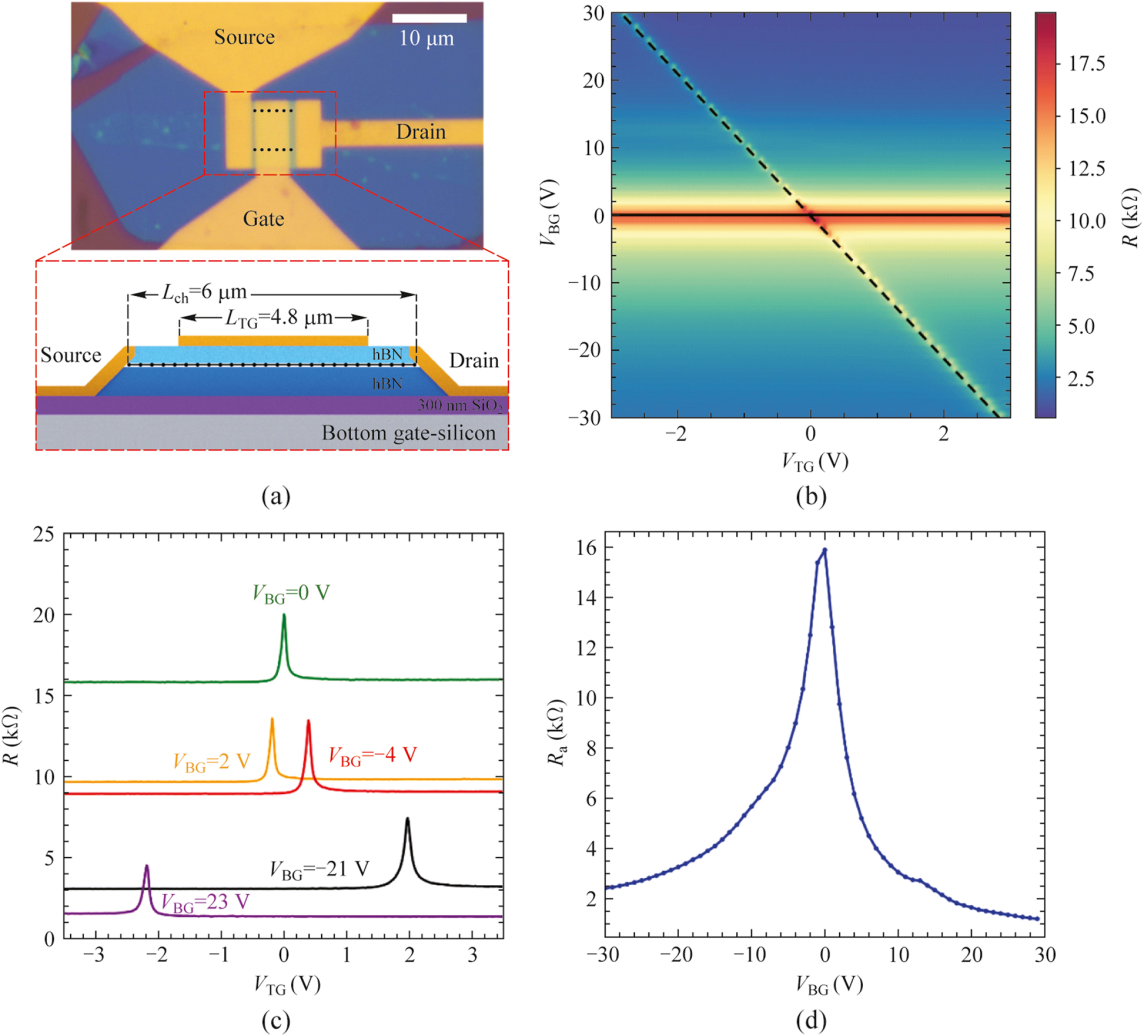
**a** Optical image (top) and lateral schematic view (bottom) of the dual gate Graphene FET. The black dotted line in the optical image indicates the edges of the graphene layer sandwiched between two h-BN flakes. **b** 2D map of the total channel resistance (*R*) as a function of the DC bias voltages applied to the top gate (*V*_TG_) and to the bottom gate (*V*_BG_) electrodes. The black horizontal (solid) and diagonal (dashed) lines correspond to the charge neutrality point (CNP) in non-top-gated and top-gated regions, respectively. **c** Total resistance as a function of the local top gate potential for five selected values of the back gate bias from panel **b**. **d** Access resistance, ${R}_{\text{a}}$, as a function of the back gate bias. Temperature was fixed at 10 K for panels **b**–**d**

Figure 1a shows the optical image of the studied graphene-FET (GFET) and a schematic lateral view of the channel structure. The GFET device consists on a monolayer graphene encapsulated between two thin hBN flakes stacked using a polymer-based method for the assembly of van der Waals heterostructures [24, 26]. The stack has been placed on top of a standard SiO_2_/Si substrate, which serves as a global bottom-gate electrode. Device fabrication, including the definition of both channel and metal electrodes, has been undertaken by using standard electron-beam lithography, dry-etching and metal evaporation techniques [24, 26]. In addition, the GFET detector contains a bow-tie antenna connected to the source and top-gate electrode as labeled in Fig. 1a to efficiently coupling the THz radiation incident on the detector. In terms of geometry, the GFET device length is *L*_ch_ = 6 µm and average width of *W*_ch_ ≈ 6 µm with a local top-gate covering only a part of the channel (*L*_TG_ = 4.8 µm). As such, there are two channel regions between drain-gate and source-gate electrodes not affected by the applied top-gate potentials. Importantly, the global bottom-gate electrode allows us to independently tune the doping level of these channel regions not covered by the top-gate and, therefore, to study their impact on the collected photocurrent in the detector. In other words, the access resistance of this device (${R}_{\text{a}}$) is tunable in our device and is given by the contact resistance graphene-metal at the source and drain electrodes plus the resistance two regions not covered by the top-gate electrode (and therefore only affected by the back-gate).

Prior to study the photoresponse of our graphene photodetector, we have measured the electrical *I* − *V* characteristics of the device in a variable temperature optical cryostat included in the THz photocurrent setup (see Supplementary Material Note 1 for more information). Figure 1b shows the device resistance (*R*) of the device, when sweeping simultaneously the top-gate voltage (${V}_{\text{TG}}$) and back-gate voltage (${V}_{\text{BG}}$) at a temperature of 10 K. Meanwhile, Fig. 1c shows the total drain-to-source resistance of the device as a function of ${V}_{\text{TG}}$ for five selected back-gate potentials from Fig. 1b. For simplicity and easier readability, the presented figures in this manuscript have been displayed as a function of the normalized back-gate voltage, i.e., ${V}_{\text{BG}}= {V}_{\text{BG}}^{*}- {V}_{\text{BG},\text{CNP}}$, where ${V}_{\text{BG}}^{*}$ is the applied back-gate voltage and ${V}_{\text{BG},\text{CNP}}$ is the back-gate voltage at the charge neutrality point (CNP). This means that in our device, the total device resistance reaches the maximum when both gates are biased such that the device is operating at the CNP potentials (i.e., ${V}_{\text{BG}}=0\text{ V}$ and ${V}_{\text{TG}}=0\text{ V}$), occurring at the crossing point of the continuous and dashed lines shown in Fig. 1b. When fixing the back-gate voltage at its CNP, the (electron or hole) carrier concentration increases in the channel region below the top-gated electrode when sweeping ${V}_{\text{TG}}$ resulting in the typical bell-shape resistance curve (see Fig. 1c, green curve) characteristic of single-gate graphene devices [21, 30, 33]. A similar result occurs when ${V}_{\text{BG}}$ is biased at fixed potentials away from ${V}_{\text{BG},\text{CNP}}$ (either positive or negative) and sweeping ${V}_{\text{TG}}$ (see Fig. 1c). However, one can see two clear main differences: First, the overall measured channel resistance curves are vertically shifted for $\left|{V}_{\text{BG}}\right|$ > 0 V as a result of the modulation of the carrier concentration in the non-top-gate regions and, second, a shift of the ${V}_{\text{TG},\text{CNP}}$ occurs as the region of the channel influenced by the top-gate electrode is also affected by the bottom gate electrode and therefore a new top-gate voltage must be applied to reach the new charge neutrality condition in this region [34].

As such, the total channel resistance, *R*, in the GFET can be understood as the sum of two different contributions [21], $R={R}_{\text{TG}}+{R}_{\text{a}}$. The first term (${R}_{\text{TG}}$) corresponds to the channel resistance below the top gated region (subjected to both local top- and bottom-gate potentials). The second contribution (${R}_{\text{a}}$) stems from channel areas non influenced by the top-gate potential. As previously introduced, this second contribution, usually known as parasitic resistance or access resistance [21, 35], is given by ${R}_{\text{a}}={R}_{\text{c}}+{R}_{\text{nTG}}$ and takes into account the sum of contact resistances (${R}_{\text{c}}$) and the channel resistance of the non-top-gated areas (${R}_{\text{nTG}}$). Both parasitic contributions limit the performance of any photodetector [21]. A good estimation of ${R}_{\text{a}}$ can be extracted from Figs. 1b and c at large doping values (high ${V}_{\text{TG}}$, see Fig. 1d), i.e., when the total channel resistance *R* is mostly independent of ${V}_{\text{TG}}$ and has a constant value (i.e., ${R \approx R}_{\text{a}}$ for large top gate potentials, see Supplementary Material Note 2 for further details about how to extract ${R}_{\text{a}}$). Moreover, from the former transport data, we can also estimate the mobility of the channel at 10 K with average mobilities exceeding 70.000 cm^2^/(V⋅s) (see Supplementary Material Note 2).

**Fig. 2:**
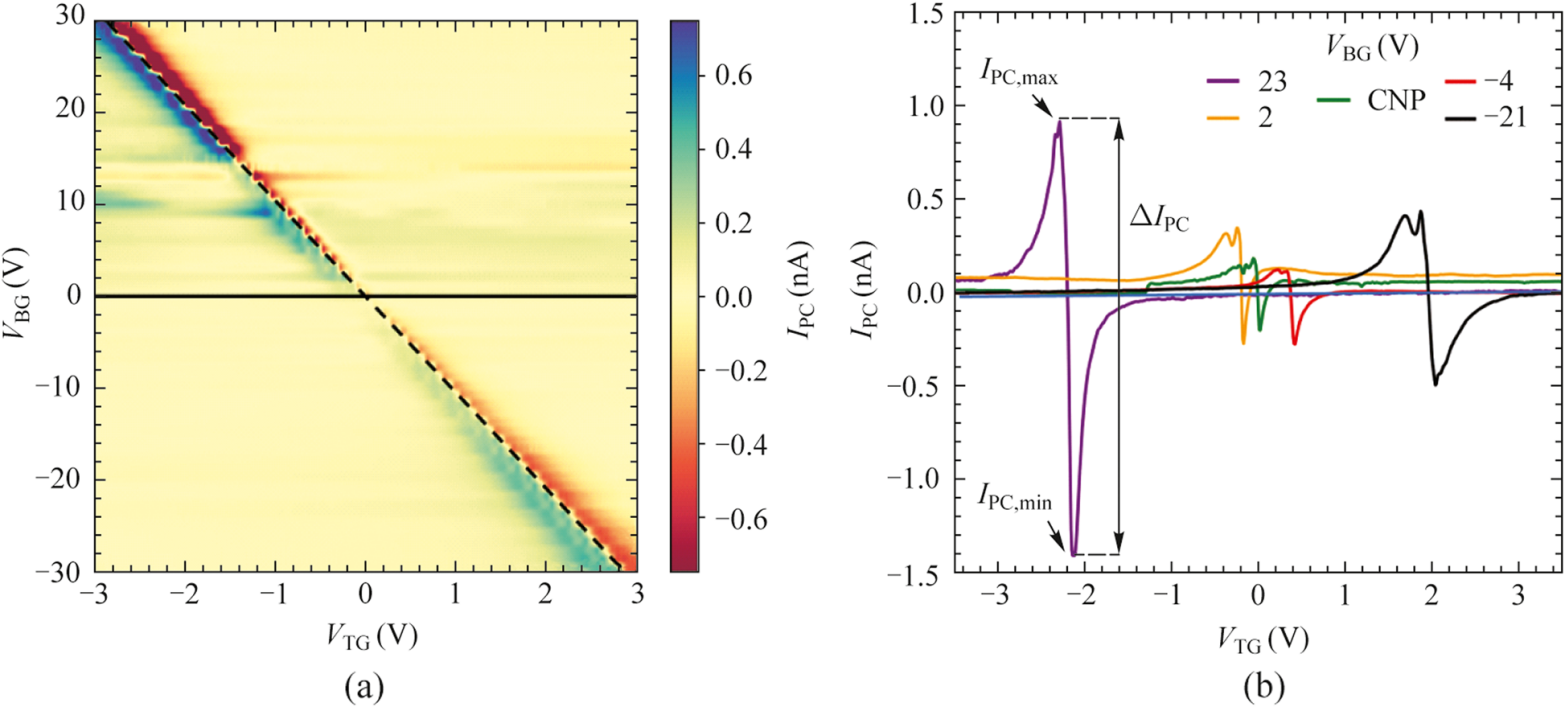
**a** Photocurrent (${I}_{\text{PC}})$ mapping as function of top gate bias (${V}_{\text{TG}}$) and back gate bias (${V}_{\text{BG}}$) for an incident radiation of 0.3 THz at 10 K. The black horizontal (solid) and diagonal (dashed) lines correspond to the CNP of the non-top-gated and top-gated regions, respectively. **b** ${I}_{\text{PC}}$ as a function of top gate voltage ${V}_{\text{TG}}$ for five selected values of a back gate bias from panel **a**

Next, we have analyzed the performance of the device when was exposed to radiation at a frequency of 0.3 THz. Figure 2a shows the evolution of the measured photocurrent, ${I}_{\text{PC}}$, with respect to ${V}_{\text{TG}}$ and ${V}_{\text{BG}}$, and Fig. 2b highlights ${I}_{\text{PC}}\left({V}_{\text{TG}}\right)$ for five selected back-gate potentials. For each fixed value of ${V}_{\text{BG}}$, ${I}_{\text{PC}}\left({V}_{\text{TG}}\right)$ exhibits an antisymmetric shape with respect to the applied top gate potential with a different sign depending on the type of carrier of the device channel as well as maximum (${I}_{\text{PC},\text{max}}$) and minimum (${I}_{\text{PC},\text{min}}$) values in the vicinity of the charge neutrality point ${V}_{\text{TG},\text{CNP}}$ for hole and electron carriers, respectively. Moreover, the actual zero-crossing occurs right at the ${V}_{\text{TG},\text{CNP}}$, and the photoresponse tends to zero at large |${V}_{\text{TG}}$| values. This qualitative behavior stems from the ambipolar charge transport presented in this material and is consistent with the Dyakonov-Shur (DS) detection mechanism (also referred to as resistive self-mixing mechanism). In this low-end THz frequency range, plasma waves injected from the source side may propagate along the channel of the FET at relatively high speed (plasma-wave propagation velocity in graphene ranges between 3 × 10^6^ and 7 × 10^6^ ms^−1^ [36]). However, despite the large propagation speed, plasma oscillations are strongly overdamped and therefore the system responds in a quasi-static manner operating in the so-called broadband (non-resonant) regime in agreement with previous studies of THz photodetectors made of graphene GFETs [24, 30, 31]. The broadband operation of our photodetector is confirmed by noting that the value of the extracted relaxation time, *τ*, of our graphene photodetector with an average mobility of 70.000 cm^2^/(V⋅s) at a carrier density of *n* ≈ 10^15^ m^−2^ is approximately 0.3 ps. At 0.3 THz, this corresponds to a device quality factor below unity, *Q* = 0.57 (*Q* = 2π*fτ*, with *f* being the incoming THz frequency), typical of a THz photodetector working in the broadband regime [13, 23, 37], in a clear contrast with photodetectors operating at higher frequencies and exhibiting resonant detection (*Q* ≫ 1) at THz fields [29, 32].

Interestingly, by examining Fig. 2 in more quantitative terms, we found that the measured photocurrent ${I}_{\text{PC}}\left({V}_{\text{TG}}\right)$ has a strong dependence with the applied back-gate bias. As the back-gate potential is positioned away from the CNP (${V}_{\text{BG}}=0\text{ V}$, solid horizontal line in Fig. 2a), we observed a substantial increment in the value of both photocurrent maxima, ${I}_{\text{PC},\text{max}}$, and minima, ${I}_{\text{PC},\text{min}}$, and subsequently the absolute difference value of $\left|{I}_{\text{PC},\text{max}}- {I}_{\text{PC},\text{min}}\right|$ in the vicinity of the CNP of the top gate potential (diagonal dashed line in Fig. 2a). In particular, we measure a maximum enhancement of $\left|{I}_{\text{PC},\text{max}}- {I}_{\text{PC},\text{min}}\right|$ at ${V}_{\text{BG}}$ > 20 V which is 20 times larger than the value at ${V}_{\text{BG}}$ = 0 V. This fact indicates that the doping level of areas not covered by the top-gate potential notably influences the photodetection and therefore the overall performance of the graphene FET device, with larger photoresponses occurring for higher doping levels.

As shown below, the observed enhancement of the THz photocurrent can be interpreted and well modeled by the formerly introduced series resistance model ${R}_{\text{a}}={R}_{\text{c}}+{R}_{\text{nTG}}$. From a theoretical perspective, the predicted DS photocurrent, ${I}_{\text{pred}}$, expected in plasmonic photodetectors when an oscillating THz field is coupled between gate and source electrodes and the device exhibit a broadband response, is given by [13, 28, 30]:

1 $${I}_{\text{pred}}=-\frac{{U}_{\text{a}}^{2}}{4}\frac{\text{d}\sigma \left({V}_{\text{G}}\right)}{\text{d}{V}_{\text{G}}},$$

where ${U}_{\text{a}}$ is the amplitude of the THz-induced ac voltage between the gate and the source electrodes, $\sigma$ is the experimental DC total conductance of the device ($\sigma = {L}_{\text{ch}}/{W}_{\text{ch}}/R$) and ${V}_{\text{G}}$ the corresponding potential at the gate electrode where the THz radiation is coupled onto the device (${V}_{\text{TG}}$ in our device). Importantly, although commonly neglected, Eq. (1) does depend on the access resistance of the actual devices, playing an important role in the overall device performance. In more detail, since $R={R}_{\text{TG}}+{R}_{\text{a}}$, the total channel conductance of the device, $\sigma$, can be expressed as:

2 $$\sigma =\frac{{L}_{\text{ch}}}{{W}_{\text{ch}} }\frac{{\sigma }_{\text{TG}}}{1 + {R}_{\text{a}}{\sigma }_{\text{TG}} },$$

where ${\sigma }_{\text{TG}}$ represents the channel conductance at the dual-gate region (${\sigma }_{\text{TG}}=\frac{1}{{R}_{\text{TG}}}$, i.e., below the top-gate electrode). To interpret precisely the observed experimental behavior of the photocurrent device (i.e., the fact that the maxima and minima values of ${I}_{\text{PC}}\left({V}_{\text{TG}}\right)$ decrease when ${V}_{\text{BG}}$ tends to ${V}_{\text{BG},\text{CNP}}$ in Fig. 2), the access resistance contribution must be introduced in the predicted photocurrent. Then, replacing Eq. (2) into Eq. (1), the predicted photocurrent when measuring as a function of the local top gate can be rewritten as:

3 $${I}_{\text{pred}}=-\frac{{U}_{\text{a}}^{2}}{4}\frac{{L}_{\text{ch}}}{{W}_{\text{ch}} }\frac{\text{d}{\sigma }_{\text{TG}}}{\text{d}{V}_{\text{TG}}}\frac{1}{{\left(1 + {R}_{\text{a}}{\sigma }_{\text{TG}}\right)}^{2} }.$$

From Eq. (3), the expected photocurrent depends on both the conductance of the graphene channel below the top-gated region as well as on the access resistance. Moreover, it can be seen that the maximum current photoresponse as a function of the local top gate potential occurs when ${R}_{\text{a}}$ is reduced (i.e., contact and non-top-gate region resistances are minimized). Such reduced ${R}_{\text{a}}$ occurs for large values of ${V}_{\text{BG}}$, in clear qualitative agreement with our experimental data (Fig. 2). Conversely, ${R}_{\text{a}}$ reaches its maximum value when the back-gate bias is set at the CNP (${V}_{\text{BG}}$ = 0 V in this research article), yielding to minimum photocurrent values at that gate-voltages (also in agreement with experiments in Fig. 2).

**Fig. 3:**
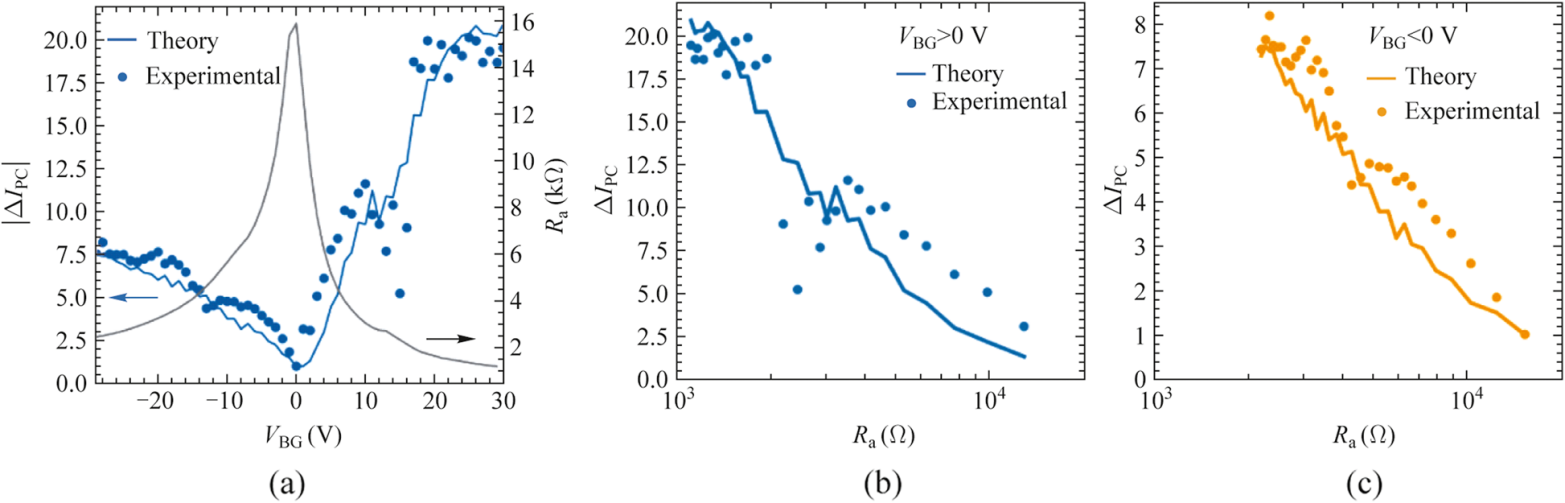
**a** Normalized ${\Delta I}_{\text{PC}}= {I}_{\text{PC},\text{max}}- {I}_{\text{PC},\text{min}}$ values (left axis) and access resistance (right axis) as a function of the back-gate voltage extracted from the measured photocurrent data (blue dots) and expected photocurrent using Eq. (3) (blue line). The normalization is carried out with respect to ${\Delta I}_{\text{PC}}$ at the back-gate ${V}_{\text{BG},\text{CNP}}$. Normalized ${\Delta I}_{\text{PC}}$ values as a function of the access resistance for positive **b** and negative **c** back-gate potentials. Solid line represents the theoretical dependence with respect to ${R}_{\text{a}}$ using Eq. (3) and dots the experimental values extracted from the measured photocurrent data. Temperature was fixed at 10 K and frequency was 0.3 THz in all panels

We further demonstrate the validity of Eq. (3) in quantitative terms by comparing the observed photocurrent dependence as a function of the applied back- and top-gate voltages (Fig. 2) with the predicted one using Eq. (3) (see Supplementary Material Note 3). Furthermore, we presented the difference between the maxima and minima of the photocurrent, ${\Delta I}_{\text{PC}}= \left|{I}_{\text{PC},\text{max}}- {I}_{\text{PC},\text{min}}\right|$ for the different applied ${V}_{\text{BG}}$ in both cases, the experimental photocurrent and the predicted photocurrent, using Eq. (3) (see Fig. 3a) and analyzed its dependence with the modulation of the access resistance. The difference of maxima and minima of the measured photocurrent (blue dots in Fig. 3a) is in excellent agreement (qualitatively and quantitatively) with the data obtained when using Eq. (3) and the DC measured values in Fig. 1 (blue line in Fig. 3a). To perform this comparison, for each value of ${V}_{\text{BG}}$, ${\Delta I}_{\text{PC}}$ has been normalized with respect to the value obtained at the CNP (i.e., ${V}_{\text{BG}}=0\text{ V}$) and therefore Fig. 3 represents the photocurrent enhancement ratio with respect to this operation point. Importantly, the maximum enhancement ratio exhibited in the system is as high as 20 when the access resistance is decreased by nearly one order of magnitude.

All these findings are summarized in Fig. 3b and c, showing the enhancement of the measured photocurrent, ${\Delta I}_{\text{PC}}$, as a function of ${R}_{\text{a}}$ when doping the channel with both electron (positive values of ${V}_{\text{BG}}$) and hole (negative values of ${V}_{\text{BG}}$) carriers. The larger photoresponse (i.e., the larger $\left|{I}_{\text{PC},\text{max}}- {I}_{\text{PC},\text{min}}\right|$) is obtained for the lower values of ${R}_{\text{a}}$ and the experimental trend is in clear agreement with the expected photoresponse using Eq. (3) (solid lines in Fig. 3b and c). In fact, we showed that the rectified photocurrent in plasmonic photodetectors at THz frequencies depends on the access resistance as $\sim a/{(1+b{R}_{\text{a}})}^{2}$, where $a$ and $b$ are parameters that are independent of ${R}_{\text{a}}$. The above presented results are essential findings for the optimal performance and guidance in the design of any state-of-the-art plasmonic THz photodetectors.

Prior to conclude, two figures of merits define the sensitivity of a photodetector, i) the current responsivity (${R}_{I}={I}_{\text{PC}}/{P}_{\text{d}}$, where ${P}_{\text{d}}$ is the incoming power at the detector) and ii) the noise-equivalent-power ($\text{NEP}=\sqrt{4{k}_{\text{B}}T\sigma }/{R}_{I}$ for a non-drain-to-source-biased detector where ${k}_{\text{B}}$ is the Boltzmann constant, and $T$ is the temperature). Then high-responsivity and low NEP values are desirable in any THz photodetector. In terms of the responsivity, it is clear that the higher measured values of ${I}_{\text{PC}}$, the better current responsivity. Thus, as we show above, an optimal performance device (i.e., the modulation of the access resistance through the back-gate bias) can increase by 20 times the rectified THz photocurrent, resulting in a direct enhancement of the responsivity by the same factor. Similarly, the modulation of the access resistance toward lower values yields to lower values of NEP (see Supplementary Material Note 4).

From application perspective, although the aforementioned analysis is shown at a cryogenic temperature (10 K), a similar behavior occurs at application-relevant, room temperature conditions where the above introduced relevant figure of merits (Responsivity and NEP) can be also enhanced by modulating and minimizing the access resistance (see Supplementary Material Note 5). While we have shown that access resistance can be easily modulated by using a dual-gate configuration, if the substrate characteristics hinder the implementation of an additional global-back-gate [38], the access resistance contribution may be minimized by reducing the gap between drain-gate and source-gate electrodes or even setting in these regions a p-type or n-type doping in graphene achieved through chemical doping [39] which will be beneficial in terms of the THz photodetector performance.

Finally, is important to remark that the performance of THz detectors depends not only on internal resistances but also on the characteristics of the photodetector’s active materials. For this reason, our study has been focused on single-layer graphene, which has been extensively studied in recent years due to its superior carrier mobility, low resistance, gapless spectrum, and ultrafast carrier dynamics. These outstanding optoelectronic properties result in the fastest response times with high responsivity at THz frequencies [40]. However, graphene is just one of many possible 2D crystals, and our findings can be extended to improve and study the performance of sensors made from other 2D materials.

For instance, among the different 2D materials, BP can support relatively high carrier mobility at room temperature (around 10^3^ cm^2^/(V⋅s)) making it suitable for the development of high-frequency plasmonic THz detectors [10, 41]. However, in contrast to graphene, BP degrades rapidly in air, affecting its structure and properties, and ultimately constrain the reproducibility of any study. Therefore, significant effort is still required to exploit its potential use in high-frequency applications.

Alternative layered 2D materials such as TMDs have also been studied in THz science due to their high modulation efficiency [9, 41] compared to graphene. However, their relatively poor mobility remains a major constraint for high-frequency plasmonic photodetectors. Additionally, their wide intrinsic band gaps (in the order of a few eVs) render them unsuitable for THz applications, which is why most studies are focused on near infrared (NIR) and visible frequencies [40].

Furthermore, topological semimetals have not been commonly considered for photodetection due to their tendency to suffer from high dark current. However, they are intriguing materials, with carrier mobility values that, in some cases, could overcome those of graphene [41]. These novel materials present a wide range of physical phenomena that could pave the way for their applications in optoelectronics and novel photodetectors at THz wavelengths [11].

## Conclusions

In summary, we have observed experimentally that the THz photoresponse in graphene FET photodetectors is notably affected by the access resistance, including metal-graphene resistances and channel areas not affected by local gate electrodes. In particular, the measured rectified THz photoresponse increases one order of magnitude in the photodetector when minimizing ${R}_{\text{a}}$, which highlights the importance of reducing any internal resistance in the device. All these findings are well captured in qualitative and quantitative terms can be captured by a series resistance model of the device. In this context, our findings, together with the optimization of i) the plasmonic antenna and external optical elements to efficiently concentrate the electromagnetic energy into the graphene channel [14], ii) low contact resistances and iii) ultra-clean van der Waals samples with high-mobility [27], would set new strategies for the development of state-of-the-art THz detectors operating at room-temperature with high optical responsivity, low values of NEP and fast response.

### Supplementary Information

Supplementary Material 1. 

## Data Availability

The data that support the findings of this study are available from the corresponding author, upon reasonable request.
