# Supplementary material for: Impact of device resistances in the performance of graphene-based terahertz photodetectors
Source: Front Optoelectron. 2024 Jun 12;17(1):19. doi: 10.1007/s12200-024-00122-6 (PMC11166907; doi:10.1007/s12200-024-00122-6)
Supplement: Supplementary file 1 — Supplementary Material 1. [file 12200_2024_122_MOESM1_ESM.pdf]

Supplementary Material: Impact of Device Resistances in the Performance of Graphene-based Terahertz Photodetectors

O. Castelló[1,2], Sofía M. López Baptista[1], K. Watanabe[3], T. Taniguchi[4], E. Diez[5], J.E. Velázquez-Pérez[1,5], Y.M. Meziani[1,5], J.M. Caridad[1,2,a)] and J.A. Delgado-Notario[1,5 a)]

[1] *Department of Applied Physics, University of Salamanca, 37008 Salamanca, Spain*

[2] *Unidad de Excelencia en Luz y Materia Estructurada (LUMES), University of Salamanca, Spain*

[3] *Research Center for Electronic and Optical Materials, National Institute for Materials Science, 1-1 Namiki, Tsukuba 305-0044, Japan*

[4] *Research Center for Materials Nanoarchitectonics, National Institute for Materials Science, 1-1 Namiki, Tsukuba 305-0044, Japan*

[5] *Nanotechnology Group, USAL–Nanolab, Universidad de Salamanca, E-37008 Salamanca, Spain*

[a)] *Author to whom correspondence should be addressed: jose.caridad@usal.es and juanandn@usal.es*

Supplementary Material Note 1: Measurement set-ups

DC transport measurements were performed using a Standford SR860 lock-in amplifier with an excitation quasi-DC current of 10nA at a frequency of 11.3 Hz. The generation of the THz wave is carried out by an RPG dual frequency THz source. This source generates a signal of 0.3 THz and 6 mW of output power using a solid-state dielectric resonator and amplifier stages based on Schottky diodes. This signal is modulated at 333 Hz mechanical chopper and them is collimated and focused on the device using an optical system. The dual gate GFET is inside a cryostat that allows us to maintain the device at a temperature between 4.5K and 300K. The window of the cryostat is made from teflon, which is transparent to THz radiation. The THz signal generates a DC current in the transistor via a nonlinear mechanism. The photocurrent was measured using a low DC input impedance current to voltage preamplifier SR570 and a lock-in amplifier SR860 whose reference follows the frequency of the chopper. Also, to apply a top and back gate bias and measure the leakage current through the gate we use a double channel Keithley 2412 source-meter. For more information, the reader is referred to previous studies[1].

Supplementary Material Note 2: Mobility estimation.

From experimental DC measurements between the gate and drain contacts as function of V_{TG} we can extract the mean mobility (μ) and account the resistance not affected by the top-gate (R_2) of electrons and holes. The total resistance of the device (drain to source) can be calculated from[2].

$$R = R_a + \frac{N_{sq}}{n_{tot} e\mu} \quad \text{(S1)}$$

where R_a is the access resistance, that corresponds to the sum of the resistance in the regions without top gate and the contact resistances (drain and source electrodes), N_{sq} is the number of squares of the graphene channel, μ is the mean mobility of the carriers in the graphene channel, and n_{tot} is the total density of carriers that can be calculated with:

$$n_{tot} = \sqrt{n^2 + n_0^2} \quad \text{(S2)}$$

where n is the charge density induced by the top gate and n_0 the residual charge carrier density:

$$n = \frac{C_T}{e}\left(V_{TG} - V_{TG}^{(CNP)}\right) \quad \text{(S3)}$$

To reduce the problem to a two-parameter adjustment, it has been used that[3]:

$$n_0 = \frac{N_{sq}}{\left(R^{(CNP)} - R_a\right) e\mu} \quad \text{(S4)}$$

Former expression that can be obtained from equation S1 assuming that n_0 correspond to the charge density at the Dirac point, where $R = R^{(CNP)}$. Combining eq S1, S2 and eq S4 we can obtain the equation described by Gammelgaard et al[3]:

$$R = R_a + \frac{N_{sq}}{\sqrt{n^2 + \left(\frac{N_{sq}}{\left(R^{(CNP)} - R_a\right)e\mu}\right)^2} e\mu} \tag{S5}$$

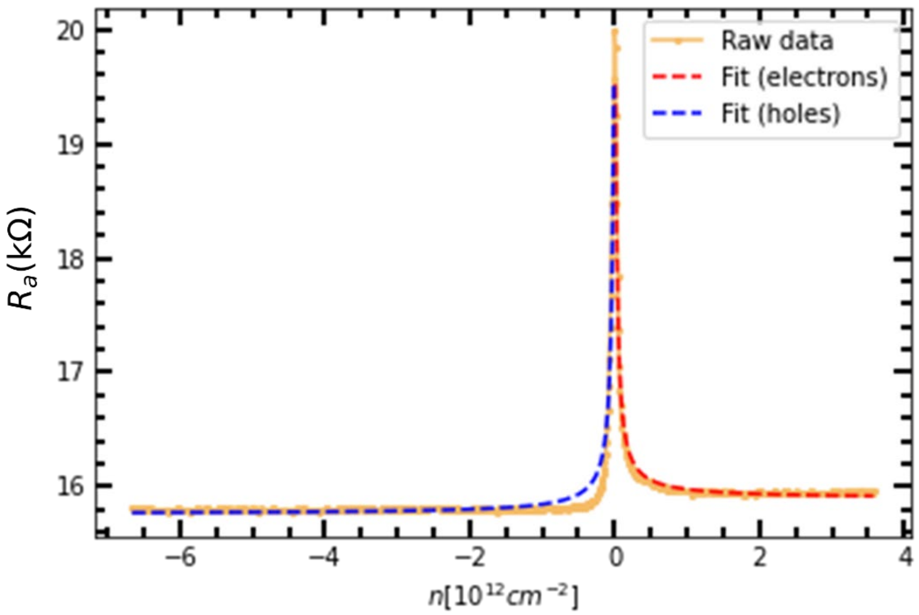

Figure S1. Experimental curve fitting of the resistance (orange line) versus carrier density for a $V_{BG} = 0V$. The red dashed line corresponds to the fitting made for the electron branch and the blue dashed line for holes.

The extracted values at 10K temperature for mobilities, μ, were around 76000 $cm^2V^{-1}s^{-1}$ and 71000 $cm^2V^{-1}s^{-1}$ for electrons and holes respectively. The extracted R_a for electrons (holes) was 15893 Ω (15756 Ω).

Additionally, as mentioned in the main text, we estimated the value of the access resistance (R_a) by recording the value of R at large top-gate potentials. Furthermore, R_a is also estimated by fitting the measured $R(V_{TG})$ curve to an expression with two free parameters R_a and μ (see equation S5). The discrepancy between both methods is rather small, below 15% (see Table S1 below) which confirm the estimation of R_a as the value of R at large V_{TG}.

	Value away from V_{CNP}		Fit to model	
V_{BG} (V)	R_a (Ω) electrons)	R_a (Ω) (holes)	R_a (Ω) electrons)	R_a (Ω) (holes)
17	1941	2349	1937	2266
30	1113	1272	1071	1203
0	15463	15297	15408	15233
-28	2557	2343	2251	2245

Table S1. Data comparison between possible access resistance extraction methods. On the left are the values corresponding to the value when we move far enough from the Dirac point (i.e. large top gate voltages) and on the right are those extracted through the adjustment procedure following equation S5 described above in this section.

Supplementary Material Note 3: Theoretical THz photocurrent.

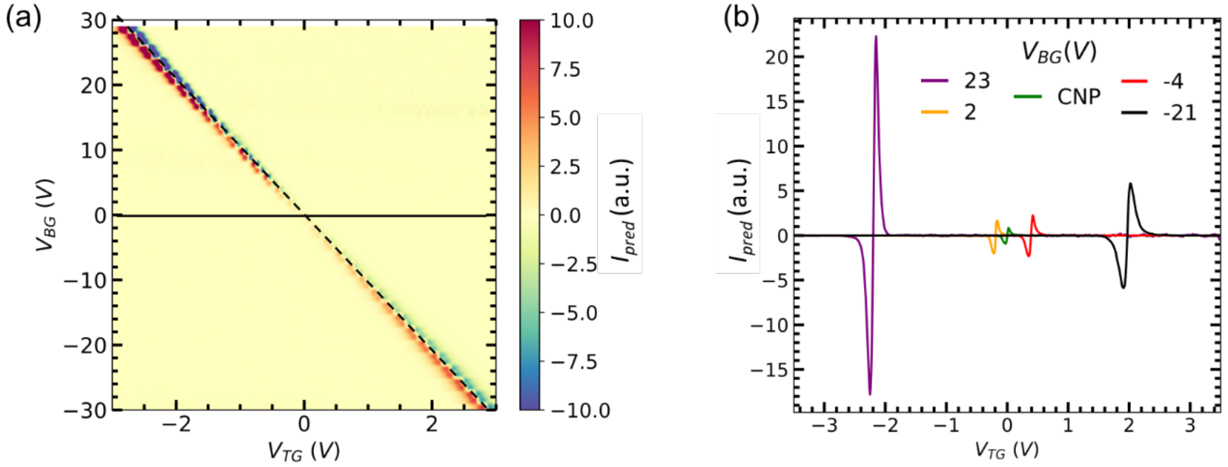

Figure S3 (a) Predicted photocurrent (I_{pred}) mapping as a function of V_{BG} and V_{TG}. The black horizontal (solid) and diagonal (dashed) lines correspond to the CNP of the non-top-gated and top-gated regions, respectively. (b) I_{pred} as a function of top gate voltage V_{TG} for five selected values of a back gate bias from panel (a). Expected values have been extracted from transport measurements at 10K following equation in the main text.

Supplementary Material Note 4: Noise-Equivalent-Power.

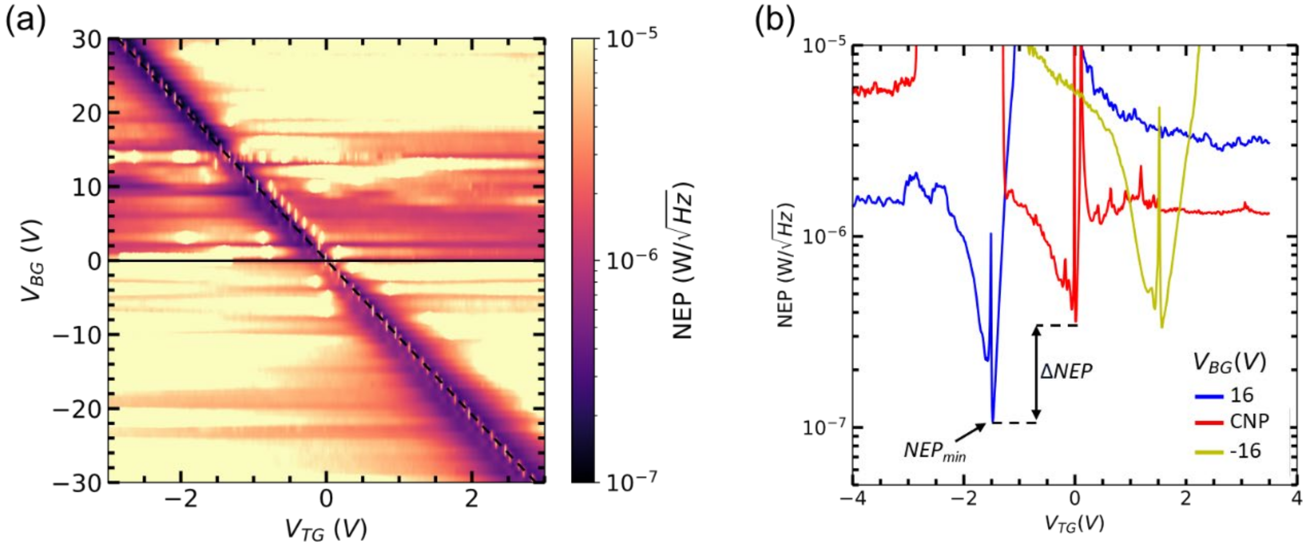

Figure S4. (a) Noise-Equivalent-Power (NEP) mapping as a function of V_{TG} and V_{BG} for an incident radiation of 0.3 THz at 10K. Horizontal (solid) and diagonal (dashed) lines correspond to the zero-charge-density states of the non-top-gated and top-gated regions, respectively. (b) NEP as a function of top gate voltage V_{TG} for three selected values of a back gate bias from panel (a). Minimizing the contribution of the access resistances yields to a reduction also in the minimum value of NEP.

Supplementary Material Note 5: Room Temperature.

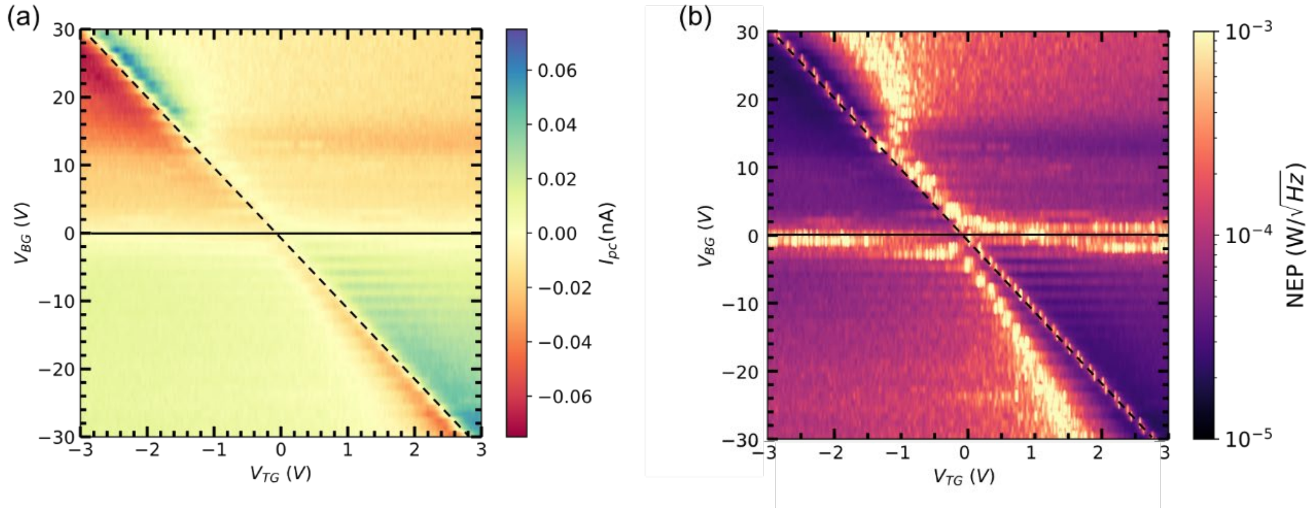

Figure S5. (a) Photocurrent mapping and (b) NEP mapping as a function of V_{TG} and V_{BG}. Both panels have been obtained for an incident radiation of 0.3 THz at Room Temperature. Horizontal (solid) and diagonal (dashed) lines correspond to the zero-charge-density states of the non-top-gated and top-gated regions, respectively.

REFERENCES

1. Delgado-Notario JA, Clericò V, Diez E, Velázquez-Pérez JE, Taniguchi T, Watanabe K, Otsuji T, Meziani YM (2020) Asymmetric dual-grating gates graphene FET for detection of terahertz radiations. APL Photonics 5:066102. https://doi.org/10.1063/5.0007249
2. Kim S, Nah J, Jo I, Shahrjerdi D, Colombo L, Yao Z, Tutuc E, Banerjee SK (2009) Realization of a high mobility dual-gated graphene field-effect transistor with Al2O3 dielectric. Appl Phys Lett 94:062107. https://doi.org/10.1063/1.3077021
3. Gammelgaard L, Caridad JM, Cagliani A, MacKenzie DMA, Petersen DH, Booth TJ, Bøggild P (2014) Graphene transport properties upon exposure to PMMA processing and heat treatments. 2d Mater 1:035005. https://doi.org/10.1088/2053-1583/1/3/035005
